# Multimodal generative AI for interpreting 3D medical images and videos

**DOI:** 10.1038/s41746-025-01649-4

**Published:** 2025-05-13

**Authors:** Jung-Oh Lee, Hong-Yu Zhou, Tyler M. Berzin, Daniel K. Sodickson, Pranav Rajpurkar

**Affiliations:** 1 Department of Radiology, Seoul National University Hospital, Seoul, Republic of Korea; 2 Department of Biomedical Informatics, Harvard Medical School, Boston, USA; 3 Center for Advanced Endoscopy, Beth Israel Deaconess Medical Center, Harvard Medical School, Boston, USA; 4 Center for Advanced Imaging Innovation and Research, Department of Radiology, New York University Grossman School of Medicine, New York, USA

**Keywords:** Machine learning, Medical imaging, Endoscopy, Magnetic resonance imaging, Tomography

## Abstract

This perspective proposes adapting video-text generative AI to 3D medical imaging (CT/MRI) and medical videos (endoscopy/laparoscopy) by treating 3D images as videos. The approach leverages modern video models to analyze multiple sequences simultaneously and provide real-time AI assistance during procedures. The paper examines medical imaging’s unique characteristics (synergistic information, metadata, and world model), outlines applications in automated reporting, case retrieval, and education, and addresses challenges of limited datasets, benchmarks, and specialized training.

## Introduction

Current unimodal AI models that interpret either text or images/videos already benefit physicians by summarizing electronic health records^1^, identifying high-risk patients for cancers^2^, and detecting lesions in medical images/videos^3,4^. However, vision-language generative AI, which integrates both linguistic and visual information, has immense potential to surpass these capabilities across the entire healthcare system, transcending specific medical fields. Techniques such as clinical report generation from images, visual question answering, and synthetic data generation are anticipated to significantly transform clinical workflows and educational practices. Nonetheless, these advancements are as of yet predominantly limited to 2D medical images. The application of multimodal generative models to high-diagnostic-value 3D medical imaging examinations, like CT and MRI, and video data, such as endoscopy or surgical video, remains in its early stages.

This perspective proposes approaches to revolutionize the interpretation and analysis of complex medical imaging data by leveraging the latest advancements in vision-language generative models, particularly video-text generative models. We argue that these powerful AI systems, originally designed for natural video understanding, possess untapped potential to transform how we process and interpret 3D medical images and medical videos. In this paper, “3D medical images” specifically refer to tomographic images from CT and MRI, while “medical videos” focus on gastrointestinal (GI) endoscopy and laparoscopy, excluding other types like 3D ultrasound or patient behavior recordings.

## Video-Text Generative/Foundation Models

Video-text generative AI is capable of creating text and videos from inputs in either modality. Foundation models^5^ within this category are trained on large multimodal datasets of videos, images, and text, making them highly versatile across various tasks. These models can generate video descriptions^6^, answer questions about video content^7^, and retrieve specific content within videos using textual queries^8^. Additionally, they are capable of tasks such as video generation^9^ and object detection/tracking^10^.

The successful integration of 2D images and text through Contrastive Language-Image Pre-training (CLIP)^11^ and its application to videos^12,13^ opened a new era for video-text foundation models. Contemporary video-text models can handle four or more modalities simultaneously^14^, including audio and subtitles in addition to videos and text inputs^15^, and can process thousands of frames at once^16^. As large language models (LLMs) gain multimodal capabilities, some video-text generative models have emerged from LLMs^17,18^. Multimodal LLMs such as OpenAI’s GPT-4o^19^, Google DeepMind’s Gemini 1.5^20^, and their successors can now perform video-related tasks, placing them within the video-text generative model category.

Labeling video data is challenging and costly, prompting the widespread adoption of self-supervised learning for training vision-text foundation models^21,22^. These self-supervised models, primarily trained with masked modeling^23^ and contrastive learning^24^ on extensive datasets, have outperformed supervised models trained on specific datasets^25–27^. The same self-supervised approach can be applied to 3D images^28^ and videos^29^ in medicine, reducing the need for expert labeling and addressing the scarcity of labeled data in the medical field.

Given structural similarities between video and 3D medical images, video-text models can be adapted to both medical videos and tomograms. However, it’s important to recognize that CT and MRI differ significantly from standard videos, and even medical videos from endoscopy and laparoscopy have distinct characteristics. These unique properties must be carefully considered when applying video-text generative models to the medical context.

## Unique Characteristics of 3D Medical Images and Medical Videos

Differences between the medical imaging data and standard video mainly arise from the specific technology and physics of medical imaging devices, as well as from the specific anatomical targets being imaged. These distinctions make it challenging to apply video-text generative models to tomograms and medical videos.

### Data Format and Video Profile

Standard video frames are typically 8-bit, 3-channel RGB images, whereas 3D medical images are usually grayscale in DICOM format, with 12-bit or 16-bit pixel ranges^30^. The broader pixel range in medical images necessitates proper windowing to enable radiologists to accurately interpret different tissue types. For instance, CT scans require different value ranges to visualize bone and lung tissues, while MRI requires specific windowing to highlight lesions, depending on the vendor and protocol used. Although deep learning models can use raw 12/16-bit DICOM images, video models pretrained on 8-bit color images/videos need proper preprocessing, such as windowing or normalization^31^.

GI endoscopy and laparoscopy videos also differ from natural videos in their color space and magnification. They often use advanced imaging techniques like narrow-band imaging (NBI)^32,33^, which enhances visibility of specific anatomical features by emphasizing certain wavelengths of light, such as those absorbed by hemoglobin, making blood vessels and tissue abnormalities more visible. In red dichromatic imaging (RDI)^34^, which uses green, amber, and red light, blood appears as dark yellowish-green, improving visibility of bleeding lesions for accurate hemostasis. The resolution and magnification of endoscopy videos may vary depending on the endoscope used and its distance from the tissue surface. Certain specialized endoscopes, when placed directly against GI mucosa, can provide up to 520x magnification^35^, capturing extremely fine details of mucosal and vascular patterns. This allows gastroenterologists to differentiate between normal tissue and dysplasia, potentially obviating the need for biopsies. Such extreme zooming is not typical in standard videos.

### Self-multimodality and Synergistic Information

Compared to videos, 3D medical images often include additional dimensions beyond the extra spatial axis, such as pulse sequence^36^ and contrast phase^37^. For example, MRI uses diverse pulse sequences like T1-weighted, T2-weighted, and diffusion-weighted images to demonstrate different tissue characteristics within the same anatomic structure. In multi-phase CT or MR examinations, multiple image stacks of the same organ are captured at different times, as the injection of exogenous contrast agents creates variations in image contrast as a function of time. Dual-source CT scans generate multiple stacks with different tube voltages. Furthermore, raw MRI or CT data can be post-processed to produce parametric maps with distinctly different information, such as signal relaxation rates or flow velocities. Consequently, 3D medical images exhibit self-multimodal properties.

Medical videos also display self-multimodality. Narrow-band imaging and red dichromatic imaging, among other techniques, can be toggled during procedures, generating different images of the same anatomy. During endoscopic or surgical interventions, clinicians may apply dyes or stains to better visualize and assess lesions, analogous to contrast phases in tomograms. Certain specialized endoscopic procedures combine multiple modalities such as ultrasound^38^, and/or fluoroscopy^39^, further demonstrating the self-multimodal properties of medical videos.

**Fig. 1:**
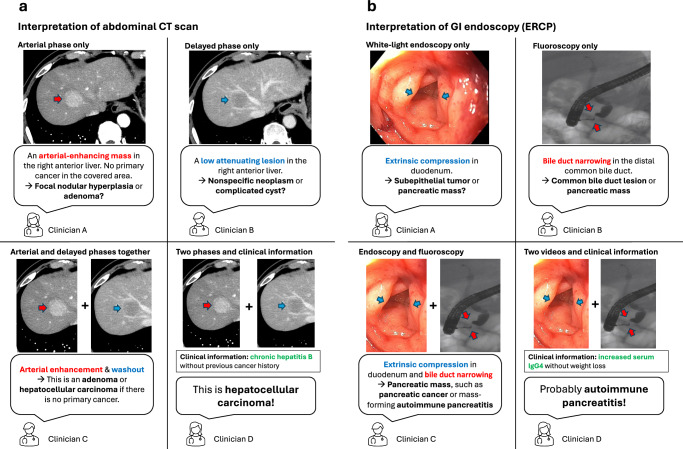
An illustrative example of the role of synergistic information in the accurate interpretation of 3D medical images and medical videos. Each panel shows how clinicians might interpret (**a**) an abdominal CT scan and (**b**) an endoscopy video (ERCP, endoscopic retrograde cholangiopancreatography) in the context of particular pieces of given information. Only when synergistic information from multiphase/multimodal images and clinical information is available (as seen with clinician D) can a clinician be confident of an accurate diagnosis.

The sequences or phases in 3D medical images contain synergistic information, making interpretation more complex than simple video analysis. Image interpretation experts can derive significant synergistic insights by analyzing all sequences or phases together, such as identifying hemodynamic features of a lesion or making differential diagnoses based on lesion composition. This synergistic information is pivotal for accurate interpretation, as illustrated in Fig. 1.

Similarly, in medical videos, integrating data from different modalities provides critical synergistic information for diagnosis. For instance, in diagnosing subepithelial tumors in the stomach, light-sourced endoscopic videos provide details on the lesion’s location and mucosal features, while endoscopic ultrasound reveals internal echo characteristics essential for distinguishing between conditions like ectopic pancreas and other subepithelial tumors.

### Metadata in Interpretation

Metadata is crucial for interpreting 3D medical images and medical videos, more so than for standard video formats. In MRI, for example, the absence of metadata detailing pulse sequences or reconstructed parametric maps—such as transit time, cerebral blood flow, or cerebral blood volume—would complicate the evaluation of MR perfusion maps^40^ in stroke patients. In colonoscopy, clinicians typically begin their detailed inspection during scope withdrawal, only after reaching the ileocecal valve^41^. This procedural metadata helps describe the approximate anatomic location of a colon polyp or the extent of mucosal involvement in ulcerative colitis. Metadata is also required in surgical videos, where experts may struggle to identify key structures or surgery phases from short clips. Moreover, since surgeries often involve procedures not fully captured on video, like vaginal procedures in laparoscopic hysterectomy^42^, metadata on surgical techniques is indispensable for accurate video interpretation.

Metadata on patient demographics also significantly impacts the interpretation of tomograms and medical videos, especially for differential diagnosis. For instance, an anterior mediastinal mass on a chest CT may suggest thymic neoplasm or goiter in an elderly patient, whereas lymphoma or germ cell tumor is more likely in a younger patient^43^. Similarly, recommendations on the same gastroscopy findings can differ based on the prevalence of gastric cancer in different populations^44^, implying the synergistic value of metadata. These examples underscore the importance of metadata in providing accurate diagnoses and interpretations for medical examinations and procedures.

### World Models

**Fig. 2:**
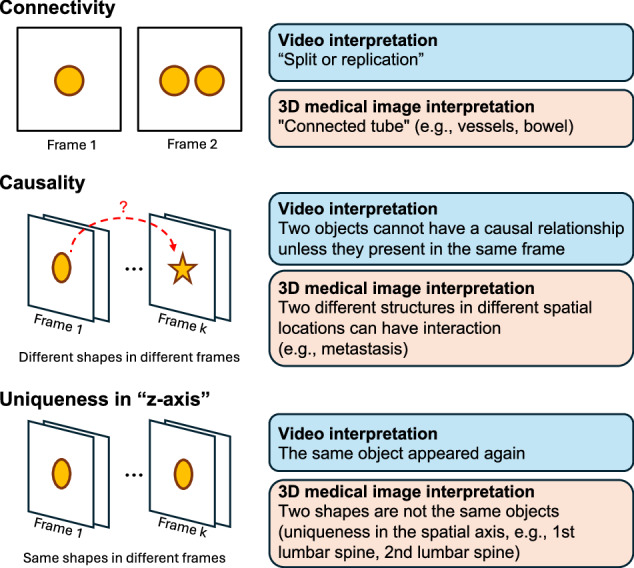
Differences in world models between videos and 3D medical imaging. (1) Connectivity: If one object becomes two identical objects in the next frame, videos may show split or replicated objects, while 3D scans capture connectivity of structures. (2) Causality: In videos, causal relationships often require co-occurrence within the same frame, but in 3D imaging, spatially separated structures can influence each other across slices. (3) Uniqueness along the z-axis: Unlike video frames, 3D volumes do not exhibit repetition of objects along the depth dimension.

A world model^45^ refers to an intrinsic system learned by AI to understand and predict the dynamics of an environment. While video models offer a promising starting point, notable differences exist between world models required for interpreting standard videos and those for 3D medical imaging. These differences primarily arise from the additional dimensions: videos add a temporal axis to 2D images, while 3D medical images add a spatial axis, possibly along with other parametric axes. Figure 2 illustrates some of the key attributes characteristic of 3D medical image models: object connectivity across frames, causality between objects, and uniqueness of similar structures.

In medical videos, a key difference lies in directionality rather than an additional spatial axis. Although the endoscope’s path might seem linear, it involves curvature and rotation, making orientation within the tubular GI lumen challenging. Even clinicians sometimes inject water into the lumen to pool in gravity-dependent areas, aiding orientation. In laparoscopy videos, navigating anatomical structures can be counterintuitive. For example, moving the laparoscope to the right in the video corresponds to the patient’s left side when imaging the upper body, but to the right side when imaging the lower body, due to the laparoscope’s insertion through the belly button. This necessitates a distinct approach to understanding orientation compared to traditional videos.

## Adapting Video-Text Models to 3D Medical Imaging

Despite the fundamental differences between standard videos and tomograms, video pre-training has been applied to 3D medical imaging tasks^46–48^. However, using video-text models for complex multimodal tasks in 3D imaging, like report generation, remains underexplored. We propose leveraging video-text generative AI to improve the interpretative capability of deep learning models in 3D medical imaging.

### Transforming 3D Medical Images into a Continuous Video

A straightforward way to adapt video-text models for 3D medical images is by converting grayscale DICOM slices to RGB and concatenating them along the “time axis” to create a long video^49^. If multiple scan windows are required due to the broader pixel intensity range of DICOM images compared to standard RGB videos, separate stacks with each window can be produced and concatenated along the time axis. Stacks of multiple sequences, contrast phases, parametric maps, or cross-sectional planes can be concatenated similarly. This method can also be applied to other types of 3D medical imaging, like PET-CT, and even multiple exams taken on different dates. By treating these concatenated slices as video frames, the dynamic capabilities of video-text models can be fully leveraged.

The key motivation for this simple concatenation method is the recent enhancement in video-text models’ capacity to process large numbers of frames simultaneously. Earlier models typically handle only 8 to 16 frames at once, but advancements in training methods and cloud computing have now enabled modern video-text models to process thousands of frames concurrently^16^. These advanced models can analyze relevant findings from text input across multiple videos, even those lasting over an hour^20^. Considering that 3D medical imaging studies often comprise several hundred slices, this capability is sufficient to accommodate not only a complete individual scan but also multiple related studies simultaneously. Consequently, modern high-capacity models can compare multiple sequential studies by processing them as a continuous video or comprehensively interpret MRI alongside additional multimodal inputs such as CT and X-ray images within a single video.

Creating a long video, rather than concatenating 3D medical images channel-wise, addresses issues like scan range variations and positional differences due to patient respiration between sequences/phases, thus enhancing the generalizability of the video-text models. For example, in liver CT scans, only the portal phase typically covers the entire abdomen to minimize radiation, while the arterial phase does not. These variations, coupled with diaphragm displacement between phases, complicate the registration of multiple sequences/phases, making channel-wise concatenation impractical.

### Beyond Visual Data: Integrating Multimodal Information

To extract synergistic information and accurately interpret 3D medical images, it’s crucial to simultaneously process multiple phases and sequences while also comprehending the medical context and metadata. Video-text models can integrate textual inputs from electronic health records, including clinical histories and lab findings, as well as metadata such as date, phase, and acquisition parameters about the 3D images^50^. In leveraging this extensive contextual information, video-text generative models derived from LLMs^51^ can significantly benefit from a knowledge base built through massive text pretraining.

**Fig. 3:**
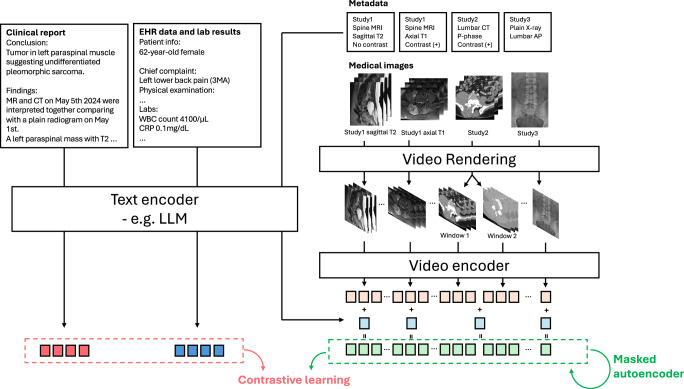
A suggested training method to adapt video-text models for 3D medical imaging. Clinical reports and electronic health records (EHR) data are input alongside multiple imaging modalities, including MRI, CT, and X-ray scans. These images can be reconfigured as a video by converting DICOM slices into RGB frames and concatenating them along the time axis, forming a long video. The video encoder processes these frames, allowing for the interpretation of complex medical imaging data. The contrastive learning approach aligns video features with text features, while the masked autoencoder aids in the training of video-text models to capture synergistic information from multimodal inputs. This method enables the comprehensive analysis of sequential studies and multimodal images within a unified framework, leveraging the enhanced capabilities of modern video-text models.

Figure 3 outlines a potential training method for video-text models integrating comprehensive information beyond traditional 3D imaging data. Unlike existing 3D medical imaging foundation models^52–54^, which typically rely only on single-phase images during training, this approach incorporates multiple image sequences alongside reports, electronic health records, and relevant metadata. Specialized training strategies, such as sequence- or phase-specific masking, can be implemented to improve the model to better understand distinct imaging phases or sequences. Furthermore, applying organ-specific masking guided by segmentation models^55^ across different imaging sequences can strengthen the model’s capability to integrate synergistic information on anatomical structures. This refined training approach may help models effectively address the unique challenges associated with interpreting tomographic images. For optimal performance, precise alignment of multimodal and longitudinal studies with their corresponding reports is essential, ensuring the models fully leverage all available data during training.

A practical pre-training approach for video-text models in 3D medical imaging is utilizing clinical reports^54,56^, which are typically paired with 3D images. However, since clinical findings in the reports often correspond to specific small volumes within the entire 3D image, using these findings as labels for the entire image can hinder accurate representation learning for video-text models. Inspired by dense captioning tasks^57^, we can decompose clinical reports into dense captions targeting specific body regions or organs. Segmentation models can then localize video frames for each organ^55^, providing detailed and localized training targets for video-text models. Moreover, video-text models capable of dense captioning can generate frame-by-frame annotations and provide visual grounding, which is essential for verifying model reliability.

## Adapting Video-Text Models to Medical Videos

Video-text models with long video inputs are particularly well-suited for analyzing medical videos, which often contain rich temporal information. For instance, during endoscopy, observing peristaltic motion in the gastrointestinal tract can provide valuable insights into digestive function^58^. Such temporal analysis is beyond the capability of non-video models. The dynamic nature of endoscopy/laparoscopy, characterized by variable lighting and the presence of fluids, bubbles, and debris, often leads to errors in traditional AI models^59^. However, video-text models with extended inputs can potentially mitigate these issues by utilizing comprehensive information across the entire videos. Additionally, combining previous and current videos as long inputs may allow tracking lesion progression or assessing therapeutic interventions over time.

Treating medical videos similarly to standard videos is a natural approach, but specific preprocessing steps may be required to handle the unique characteristics of endoscopy and laparoscopy data. These steps may include color space conversion^60^, image enhancement^61^, and removal of specular reflections caused by the endoscope light source^62^. For labeling, a dense captioning approach^63^ can be applied to process clinical reports to create captions for medical videos, focusing on specific anatomical landmarks, lesions, or abnormalities observed during the endoscopic or surgical procedures.

A critical factor to consider when implementing video-text generative models in medical videos is real-time interaction with a physician. Unlike 3D medical images, which are generally captured in a predetermined protocol and interpreted after patient encounters, medical videos can be influenced by the physician’s decisions and AI assistance during the procedure. For example, the gastroenterologist or surgeon may extend inspection time in critical areas identified by AI. Real-time AI support can aid live clinical decisions, such as whether to biopsy or resect lesions, necessitating swift interaction between the AI model and a physician. Given the live, interactive nature of endoscopy and surgery videos, inference speed is crucial. Recent advancements in vision-language generative AI, such as GPT-4o, have shown the potential for real-time interaction.

**Fig. 4:**
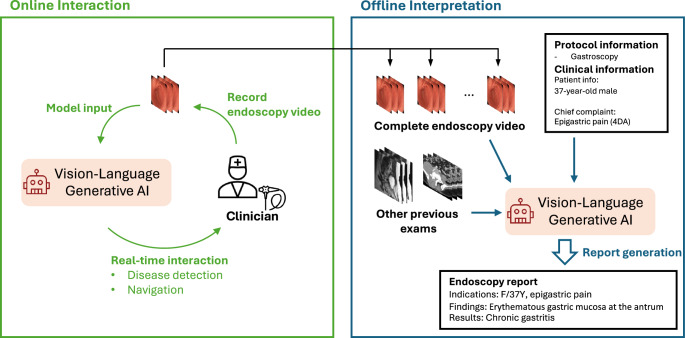
A clinical application of vision-language generative AI in endoscopy. Through real-time interaction with physicians, the vision-language AI assists in ensuring no lesions are missed. After a physician records a whole endoscopy video, the AI generates a comprehensive report that incorporates the endoscopy protocol, the patient’s clinical history, and findings from previous exams such as CT and MRI scans.

As discussed earlier, metadata is essential for interpreting endoscopic and laparoscopic videos. Protocol data and clinical information should be provided to enhance the accurate interpretation of these videos. The potential application of a video-text generative model considering these factors is illustrated in Fig. 4.

## Opportunities and Applications

The impact of video-text generative models on clinicians’ workflow could be extensive and profound, enhancing both efficiency and patient outcomes. These models can automatically draft preliminary reports for both 3D medical images^56^ and medical videos^64^, significantly reducing the time clinicians spend on documentation. Such initial drafts can facilitate emergency triage, enabling timely intervention through prompt alerts to clinicians. Moreover, workflow phase recognition for surgical and endoscopic video may enable post-hoc analytics focused on measuring efficiency and quality of procedures while providing constructive feedback to clinicians^65^. Generative models may also offer real-time guidance to augment procedural performance and reduce cognitive load during challenging endoscopic or surgical tasks, for instance by highlighting safe planes of tissue dissection^66^, or by automatically halting cautery current if an electrosurgical knife is inadvisably close to a critical structure.

Video-text retrieval techniques powered by multimodal AIs can assist clinicians to quickly search for similar cases in a database of tomograms or medical videos, facilitating comparative research and aiding in the diagnosis of rare or challenging cases. This technology can also improve communication among clinicians. A common challenge in medical data is correlating written findings with the images without proper annotation. Traditionally, this requires consultation with specialists. Video-text generative models can streamline this process by displaying relevant image slices or video frames corresponding to the written report and offering question-answering functionalities for further clarification.

These video-text generative models can also create educational content based on specific diagnoses or textual descriptions. Modern text-to-video generation models have demonstrated a remarkable ability to simulate realistic and temporally coherent videos from text inputs^67,68^. These synthetic surgical or endoscopy videos and simulated 3D medical images can serve as valuable training tools for a wide range of clinicians while preserving patient privacy.

The learned representations from training video-text generative models on 3D medical images or medical videos can have broader applications beyond their primary uses. These representations, which capture 3D anatomical relationships and multimodal correlations, could enhance diagnostic accuracy even with limited or low-quality imaging data, such as from abbreviated exams or point-of-care devices.

Finally, recent advances in scaling test-time compute^69^ have substantially boosted the performance of vision-language models across various benchmarks^70,71^. These improvements in reasoning capabilities provide promising solutions for tackling the inherent complexity of interpreting 3D medical images and medical videos. Since 3D medical images and videos are typically voluminous, with clinically relevant details often distributed in complex patterns across the data, models with advanced reasoning abilities can more effectively identify and integrate these scattered findings. This capability substantially increases the practical utility of video-text models in clinical settings, particularly for diagnostic tasks requiring sophisticated integration of spatial and temporal information. Thus, reasoning models hold significant potential for bringing the clinical applications discussed above closer to real-world implementation.

## Challenges and Future Directions

The primary challenge in adapting video-text models to 3D medical imaging and medical videos is data scarcity. While self-supervised methods for video-text models have advanced, there are only a few small open-source datasets^72,73^ suitable for 3D medical images and medical videos. For example, a recent relatively large video-text dataset contains seven million videos with 234 million video clips^74^, whereas CLIP on 2D images was trained on 400 million paired image-text pairs^6^. This highlights the lack of equivalent datasets for 3D medical images. A practical approach, therefore, involves pretraining video-text models on existing video-text datasets and then fine-tuning them on 3D medical data or medical videos^51,75^. Concurrently, global efforts to create and share more open-source datasets are crucial.

Various vision-language models have emerged in the medical field, including BioMedGPT^76^, MedPaLM^77^, LLaVa-Med^78^, and MedVersa^79^. However, most of these models cannot adequately handle multiphase 3D images or long medical videos, and none utilize multiple phases or sequences simultaneously during training. Our proposed training methods could help these models develop robust interpretive capabilities, but implementation requires carefully curated datasets containing complete image sequences and longitudinal studies. However, such comprehensive datasets raise legitimate privacy concerns, as 3D medical images can be reconstructed into recognizable shapes, and the inclusion of multiple time points increases the risk of patient re-identification. Possible solutions include institution-level deidentification, as used in MIMIC dataset^80^, and mild distortion of surface areas to protect patient privacy.

The field also lacks suitable datasets for downstream tasks and benchmarks to evaluate model performance in interpreting 3D medical images and videos, especially regarding synergistic information and unique world models. Without these resources, we cannot properly assess how video-text models fail in medical image and video interpretation. Suggesting differential diagnosis and lesion localization represent promising downstream tasks to verify proper interpretation of synergistic information. For example, distinguishing between liver hemangioma, adenoma, and focal nodular hyperplasia, or differentiating between brain abscesses and cysts on MRI, requires models to integrate information from multiple phases and sequences. Similarly, accurate lesion description in medical videos demands a sophisticated world model from video-text models.

While reasoning models show promise in this domain, appropriate reasoning datasets to train them do not yet exist. Fortunately, radiologic and endoscopic/laparoscopic reports already contain substantial reasoning information, potentially simplifying the construction of such datasets. This approach may offer the most direct path to enhancing the interpretive performance of video-text models.

Beyond the unique characteristics previously discussed, further complexities arise when adapting video-text models to 3D medical imaging and videos. Clinical reports paired with the medical data often contain varied information such as comparisons with prior studies or different types of exams, complicating the training process. Moreover, real-world tomograms can also include reconstructed volume images and dynamic videos. Techniques like cerebral CT angiography and MR cholangiography generate 3D visualizations of anatomical structures from various angles, while cardiac MRI captures heart motion in video sequences. These added complexities necessitate continuous research to enhance the training methodologies for video-text generative AI models in the medical field.

## Conclusion

The successful application of video-text generative models in medicine can revolutionize clinical workflows, enhance diagnostic accuracy, facilitate clinician-clinician communication, and provide valuable tools for education and training. Despite this promising potential, several challenges must be addressed. These include the limited availability of large-scale, open-source datasets suitable for self-supervised learning, the complexity of training models on data containing synergistic information, and the engineering hurdles associated with the unique structures of 3D medical images and medical videos. To overcome these barriers, future research should focus on creating comprehensive datasets that preserve patient privacy, developing benchmarks to evaluate models’ capabilities to integrate multi-sequence information, and advancing training methodologies tailored specifically to the complex multimodal characteristics of 3D medical imaging and medical videos.

## Data Availability

No datasets were generated or analyzed during the current study.
